# Knowledge exchange sessions on primary health care research findings in public libraries: A qualitative study with citizens in Quebec

**DOI:** 10.1371/journal.pone.0289153

**Published:** 2023-07-25

**Authors:** Maude Laberge, Francesca Katherine Brundisini, Hervé Tchala Vignon Zomahoun, Jasmine Sawadogo, José Massougbodji, Amédé Gogovor, Geneviève David, France Légaré

**Affiliations:** 1 Faculty of Medicine, Department of Social and Preventive Medicine, Université Laval, Quebec, Canada; 2 VITAM Centre de Recherche sur la Santé Durable, CIUSSS de la Capitale Nationale, Québec, Canada; 3 Centre de Recherche du CHU de Québec-Université Laval, Université Laval, Québec, Canada; 4 Quebec SPOR-SUPPORT Unit, Quebec, Canada; 5 Tier 1 Canada Research Chair in Shared Decision Making and Knowledge Translation, Quebec, Canada; 6 Department of Family Medicine and Emergency Medicine, Université Laval, Quebec, Canada; 7 First Nations of Quebec and Labrador Health and Social Services Commission, Quebec, Canada; 8 Centre d’excellence sur le Partenariat avec les Patients et le Public, Centre de Recherche du CHUM, Québec, Canada; 9 École Nationale d’administration Publique, Québec, Canada; University of Verona, ITALY

## Abstract

Little is known about knowledge transfer with the public. We explored how citizens, physicians, and communication specialists understand knowledge transfer in public spaces such as libraries. The initial study aimed at evaluating the scaling up of a program on disseminating research findings on potentially inappropriate medication. Twenty-two citizen workshops were offered by 16 physicians and facilitated by 6 communication specialists to 322 citizens in libraries during spring 2019. We did secondary analysis using the recorded workshop discussions to explore the type of knowledge participants used. Participants described four kinds of knowledge: biomedical, sociocultural beliefs, value-based reasoning, and institutional knowledge. Biomedical knowledge included scientific evidence, research methods, clinical guidelines, and access to research outcomes. Participants discussed beliefs in scientific progress, innovative clinical practices, and doctors’ behaviours. Participants discussed values related to reliability, transparency, respect for patient autonomy and participation in decision-making. All categories of participants used these four kinds of knowledge. However, their descriptions varied particularly for biomedical knowledge which was described by physician-speakers and communication specialists-facilitators as scientific evidence, epidemiological and clinical practice guidelines, and pathophysiological theories. Communication specialists-facilitators also described scientific journalistic sources and scientific journalistic reports as proxies of scientific evidence. Citizens described biomedical knowledge in terms of knowledge to make informed decisions. These findings offer insights for future scientific knowledge exchange interventions with the public.

## Background

Despite the growing body of evidence, research production has not automatically led to such knowledge being translated effectively in everyday decision making [1–5]. This divide is often described as the "evidence-to-practice" gap [4,6,7]. Researchers, decision-makers, managers, health practitioners and patients have started to develop effective knowledge translation (KT) strategies to bridge this gap [2,4–8]. Over time, KT approaches are bound to be continuously adapted and evolving to address the diverse barriers to uptake of research results in decision making [2,9,10]. Earlier KT models focused on passive, pre-packaged, and one-way transmission of knowledge from researchers to its users (i.e., the push approach) [9,11,12]. In contrast, other KT approaches have been ingrained in purely user-centric approaches (i.e., the pull approach) within which users of knowledge require actively the new knowledge being produced by researchers. However, in recent years, KT approaches have increasingly integrated knowledge production and dissemination in a transactional and interactive space defined by its users [9,11,12]. Literature has shown that interactional methods are more effective than traditional passive methods (either pull or push alone) [8,9,12]. As demonstrated by interactive learning approaches, engaged scholarship and community-based participatory research, individuals with meaningful interactions can learn and gain knowledge more effectively [2,12,13].

Today, knowledge transfer emphasizes the active dissemination of knowledge by targeting and interactively involving users in knowledge exchange activities, such as small group learning activities with end-users [9,14]. In primary care services, users of primary care research outputs are both health professionals providing care and the public receiving such care [3,4,6]. While many strategies address the gap between researchers and health professionals [6,7,15,16], little is known about interactive knowledge transfer between researchers and the public, i.e., the beneficiaries of care [17,18]. So far, conventional transfer of knowledge has been carried out by health care professionals in the context of individual clinical consultations, from health professionals to patients and/or their relatives. Mass media can also transfer knowledge between researchers and the public, but the interactive dimension is generally non-existent. Instead, public libraries are excellent partners for health policymakers, health practitioners, and the public to improve public health [19–23]. They have a great population outreach and are well-positioned to engage and educate the community as public libraries play a key role in supporting literacy as well as health literacy [21–23].

Only in Quebec in 2015, public libraries served about 96.2% of the population, with about 1.5 million Quebecers participating in activities offered by their public library [24].

Previous work fostered exchange and dissemination of research results between researchers, clinicians, and the public [24]. The results of this evaluation were promising in terms of effectiveness to improve the public’s knowledge on the topic of interest, the feasibility, and acceptability of these activities by the target users of the research results (citizens), but also by the producers of new knowledge as well as the mediators between both (clinicians, journalists, librarians). More specifically, it was observed that future knowledge transfer interventions with the public should involve all relevant stakeholders in the development of dissemination activities; second, that additional time should be accounted for and scheduled for the selection of the topic of interest for the public; that more time should be allocated for the exchanges between the speakers and the participants for data collection purposes; and that financial compensation of physician-speakers and communication specialist-facilitators and the purchase of audio-visual materials should be included in the project’s budget.

Given the gap in knowledge in the KT field presented above, we explored how different stakeholders understand knowledge in knowledge exchange sessions held in public libraries. More specifically, we explore: How do members from the public, communication specialists, and physician-speakers understand, conceptualize, and accept knowledge in knowledge exchange sessions conducted in public libraries?

## Methods

This study is part of a parent project aimed at designing, implementing and evaluating the scaling up of an effective pilot program on disseminating research results in public libraries using an integrated knowledge translation framework [25]. While the parent study’s overall goal aimed at evaluating participants’ acceptability, appropriateness, and knowledge gain, we decided to use the data gathered from the citizen workshops’ discussion sessions to explore participants’ understanding of knowledge. To do so, we conducted a secondary post hoc analysis based on transcripts produced in the parent research project [26–28]. Therefore, the following section will be divided into two parts: (a) the parent study and (b) the secondary post hoc analysis.

### Parent study

#### Aims and study design

The parent study was a retrospective pre-post intervention study aimed at scaling and evaluating a dissemination intervention with the public in public libraries in the cities of Québec and Montreal [24]. The parent study’s intervention grounded its design on the Integrated Knowledge Translation framework (IKT) [14,29,30]. This framework recognizes that knowledge is a value-laden social product developed through a dynamic, non-linear, and collaborative process with those who use it [14,29–32]. Following the IKT framework, the parent study’s research team purposively recruited targeted knowledge users as research partners, including a patient partner (i.e., a patient or informal caregiver trained in research practices), public library officials, communication specialists, physicians, and researchers. Together, the research team and the targeted knowledge user representatives jointly co-developed and operationalized an overarching dissemination and knowledge exchange strategy designed for the public defined as "citizen workshop". To do so, they all participated and developed the components of the project, that is, the selection of the research findings to disseminate; the development of the citizen workshop’s content and format, including the patient video testimony; the actualization of the citizen workshop in public libraries; and the citizen workshops’ evaluation. The topic of potentially inappropriate medication was selected because it was determined to concern more people than if the topic had been condition-specific. The thought was that it would attract more people to the workshops.

#### Study intervention, sample and settings

Citizen workshops are public interactive workshops aimed at disseminating and exchanging empirical research knowledge to citizens in public settings. Each workshop–entitled "Des medicaments en trop?" (i.e., «Too many medications?")–comprised a computerized 45-minute slide deck presentation that was presented by a physician and facilitated by a communication specialist in French. The slide deck presentation also included a video of the patient partner providing her experience related to the research findings presented during the workshop. This presentation was followed by a 45-minute knowledge exchange session between the physician-speaker, the communication specialist-facilitator and the public. The knowledge disseminated and exchanged in the workshops focused on selected primary care research outcomes addressing the topic of potentially inappropriate medications (PIMs) among the elderly [33]. The research team of the parental study conveniently recruited 18 physicians to present the selected findings and six communication specialists to facilitate the workshops. A total of 362 adult citizens voluntarily participated at the workshops. Overall, 27 citizen workshops were held from April to May 2019, in 26 public libraries that agreed to participate in the research project in Montreal and Quebec City. Each city’s director of libraries played a key role in inviting all the libraries in their respective cities to participate in the project. Selection was based on a convenience sample, with libraries that had the required amenities (ex: space) and the availability to hold the workshop in the Spring of 2019. Although we did not collect information on the specific profile of each library’s patrons, there was a diversity in their location, some serving larger proportion of immigrants and older adults, yet limited to urban settings.

#### Data collection

The parent study’s research team audio recorded 22 out of 27 of the workshops’ knowledge exchange discussions, i.e., the session after the slide deck presentation [25]. Five recordings were lost due to technical issues during the workshops. The final 22 recordings were transcribed verbatim using a commercial service and validated by one of the authors (JS). Citizen participants’ sociodemographic characteristics such as age, sex and highest level of education were also collected. The parent study also collected other data from self-administered questionnaires with closed and open-ended questions and direct observers’ notes in predefined data grids on the workshop’s settings and format characteristics.

#### Ethical considerations

Participants provided a verbal consent, which was aligned with the ethical approval received. To know the number of participants in each workshop, we used an attendance sheet. We gave a brief oral presentation of the project and distributed an information sheet about the research project and two evaluation questionnaires with predetermined unique numerical codes. We also specified that the workshop was going to be recorded and that any participant who does not wish to be recorded may decide to withdraw or not participate in the discussion. Consenting participants completed the questionnaires anonymously and participated in the discussion.

The study was approved by the research ethics committee of the *Centre universitaire intégré de santé et services sociaux (CIUSSS) de la Capitale-Nationale* [Project 2017-2018-19]. The committee noted that the project was of minimal risk and that there was no need to present a written consent form to participants.

### Secondary post hoc analysis

We conducted a secondary analysis study using interpretive description. Secondary post hoc analysis refers to the use of data that has not been collected by the analyst [34]. It is common and often used among historians, epidemiologists, and economists, yet considered relatively new in qualitative research [34,35]. We selected this approach because it allowed us to use data on knowledge transfer activities with the public that would be otherwise difficult to collect. The parent study is one of the first and only large-scale knowledge transfer implementation studies to disseminate knowledge to citizens in public settings. We conducted this study after the completion of the parent study data collection and analysis. At that time, three researchers with qualitative research experience joined the project to conduct the post hoc analysis study. All members of the original research team participated in this secondary post hoc study to maintain the integrated knowledge translation approach of the parent study.

In addition, the team members’ knowledge and familiarity with the parent study provided valuable insights on the study’s context and setting. In this post hoc analysis study, we adopted a pragmatic epistemological stance and constructivist worldview [36]. More specifically, we employed an interpretive description methodology to explore the type of knowledge participants used during the workshops’ discussion sessions, after the slide deck presentation [36–39]. Traditionally used in nursing to inform clinical practice, researchers have used interpretive description across applied health practices and management, teaching, and health policy analysis [39–44]. We selected this methodology because it allows us to descriptively explain a phenomenon to generate knowledge for practice [36,38,39]. For us, interpretive description was useful because we aimed to capture a range of concepts from those who participated as pragmatic insights that inform the practice of knowledge exchange with the public [36,39]. To do so, we looked at what meanings and interpretations participants attach to knowledge discussed in public knowledge exchange sessions. Finally, interpretive description uses a logic of general qualitative research principles [36,38,45]. This way researchers can pragmatically answer questions addressing complex phenomena in an iterative and emergent way [36].

#### Data characteristics

Our secondary analysis used clean, uncoded transcripts from 22 citizen workshops administered and evaluated in the parent study [25]. After reviewing all available data, we determined not to use the data collected from the open-ended questions in the self-administered questionnaires or the direct observers’ notes, as that data did not allow us to answer our research question. We decided to use the transcripts as we were interested in the dialogical nature of the discussions held during the workshops, i.e., the non-hierarchical, symmetrical, and two-way approach to communication. We viewed these discussions as a joint effort among equal partners to seek true understanding and knowledge [46]. Although these group discussions did not follow predetermined questions, the initial information session delivered by the physician-speakers and communication specialist facilitators through the presentation on PIMs guided participants’ discussions while leaving room for emergent opinions and beliefs [47]. In addition, the communication specialist made sure the environment was respectful, and all participants participated comfortably and ensured that people stayed on topic, putting the audience’s questions into perspective, and rewording the doctor’s answers when necessary. Based on the available transcripts collected in the parent study, sixteen physician facilitators, six science communication facilitators, and 322 citizens participated in the study across 22 public library citizen workshops. Of the citizen population, 70 were male, and 198 were female (Table 1). The mean age was 64.7 years of age, and most had completed post-secondary education (Table 1). On average, 14 citizens participated in each workshop. Most physician-speakers and communication specialist-facilitators were female, with only four physician-speakers and two communication specialist-facilitators being male. Due to difficulty in recruiting physicians, the invitation was extended to family medicine residents. There was a total of 16 physicians and 2 residents. They were younger than Quebec’s physician population and were all able to communicate fluently in French.

**Table 1:** Citizen participants’ characteristics.

Characteristics		N (%)
**Sex**	Female	198 (70.5)
	Male	70 (24.9)
	Missing data	13 (4.6)
**Age (years)**	Mean age (SD)	64.7 (12.5)
	Missing data	15
**Highest educational level**	Secondary or lower	46 (16.4)
	College	67 (23.8)
	University	153 (54.4)
	Missing data	15 (5.3)
**Total**		281*

*281 participants out of 322 have completed the sociodemographic questionnaire.

#### Data analysis

One author (FKB), a qualitative researcher, performed the analysis of all transcripts. A second researcher (AG) analyzed 10% of the transcripts. FKB regularly met and discussed the coding process and findings with AG and two researchers (HTVZ and ML) familiar with the overall project. The coded data was then iteratively discussed and interpreted by all the members of the team. We analyzed the text in French and produced analytical results in English. Data were managed and analyzed using Nvivo^®^v.12 qualitative analysis software [48]. The analysis followed an inductive and iterative two-staged thematic analytical approach similar to grounded theory: a first open-coding descriptive stage, where FKB and AG stayed very close to the data (i.e., indent-by-indent coding) to describe the data’s characteristics; and a second interpretative focused-coding stage, in which FKB and AG adopted the constant comparison method [49]. FKB systematically drafted analytical memos to enhance analytical sensitivity and refine the initial descriptive codes into abstract categories and themes [36,49]. As the analysis moved forward, the team discussions of the coding process and emergent concepts helped develop new interpretative insights leading to a more refined analysis. As suggested by Thorne, interpretative description began on the grounds of analytical, "informed questioning" [36,39]. Our data analysis was guided by the sensitizing concepts of epistemological values as identified in Tonelli [50] and Parker et al. [51], and the foundational concepts of Kleinman’s multilayered explanatory model [52]. Epistemological values and culturally patterned social and personal views of sickness and health enabled us to identify participants’ preferred sources of knowledge and ways of thinking about knowledge [49–52]. FKB also remained engaged with the data for an extended time and ensured to provide thick descriptions and audit trail of the data [36]. Finally, the researchers analyzing the transcripts practiced reflexivity throughout the analysis process, that is, the practice of continually reflecting on their perspectives in the study [53,54]. Before starting the analysis, FKB and AG wrote a document setting out their previous research experiences and perspectives on their understanding of knowledge to understand their preconceptions and how they might affect their data interpretation. They referred to this document during the analysis process. All researchers had no relationship with citizen participants, and FKB and AG, who conducted the analysis, had no relationship with the physician and communication specialists. Finally, trustworthiness was also established by debriefing with the co-investigators of the parental study throughout the whole research process.

## Results

Citizens, communication specialists-facilitators, and physician-speakers distinguished between biomedical knowledge, sociocultural beliefs, value-based reasoning, and institutional knowledge when discussing primary care research findings on PIMs during public libraries citizen workshops. Our findings are summarized in Table 2. In the following sections, we use quotes from the dialogues to illustrate the key concepts described by the participants.

**Table 2:** Summary of perspectives and descriptions of the four kinds of knowledge.

Kind of knowledge	Physicians	Communication specialists-facilitators	Citizens
*Biomedical Knowledge*	scientific evidence, epidemiological and clinical practice guidelines, and pathophysiological theories	scientific evidence, epidemiological and clinical practice guidelines, and pathophysiological theories scientific journalistic sources	knowledge to make informed decisions
*Sociocultural Beliefs*	based on reports of individual cases (from professional experiences in clinical settings, expert opinions that included medical expert consensus, consultation and clinical experience of other health professionals)	based on reports of individual cases (professional experience as scientific journalist, scientific media)	based on reports of individual cases (own experience, family, friends) Perceptions of physicians’ qualifications and influence from the pharmaceutical industry
*Value-based Knowledge*	respecting and empowering patients to gain a greater role and become active participants in health care; thrust in terms of credibility and accountability	respecting and empowering patients to gain a greater role and become active participants in health care; thrust in terms of credibility and accountability	patient autonomy, ensuring patient empowerment and partnership through education and fair participation in decision-making; trustworthiness in terms of transparency and the legal and ethical conduct of health professionals
*Institutional Knowledge*	trustworthy sources of knowledge from traditional and online outlets, such as conferences, Doctissimo, Extenso, Passeport Santé, and tools, such as Carnet Santé Quebec and Choosing Wisely; systemic barriers to care delivery	trustworthy sources of knowledge from traditional and online outlets, such as conferences, Doctissimo, Extenso, Passeport Santé, and tools, such as Carnet Santé Quebec and Choosing Wisely; systemic barriers to care delivery	interplay of relationships among institutional actors and material resources embedded in practices, routines, and procedures; lack of coordinated care and lack of knowledge and skills to navigate the health system

### Biomedical knowledge

Physician-speakers and communication specialists-facilitators understood biomedical knowledge as scientific evidence, epidemiological and clinical practice guidelines, and pathophysiological theories. These participants shared a common understanding of scientific evidence as systematically collected, analyzed, and synthesized evidence according to rigorous and standardized research methods. Scientific evidence mainly addressed fundamental and applied health research outcomes derived from experimental, observational, and meta-analysis studies. Communication specialists-facilitators also described scientific journalistic sources and scientific journalistic reports as proxies of scientific evidence. Both physician-speakers and communication specialists-facilitators also understood biomedical knowledge as epidemiological and clinical practice guidelines, standards and tools developed through research to inform prescription and treatment decision-making. As one physician exemplified: “Yearly exams in adults have clearly been proven, there are large meta-analyses that have been done by reliable sources of studies where names, where we know that we do not reduce mortality in the population by doing yearly exams.” (Workshop 13)

And similarly, a communication specialists-facilitator described scientific knowledge as based on research studies: “Then, in the Lanaudière study, it was also noticed that first generation antihistamines, such as Benadryl, are prescribed.” (Workshop 18)

Finally, these participants expressed biomedical knowledge as pathophysiological theories derived from empirical evidence. These theories addressed the causes, effects, and consequences of medications and were often used in combination with diagnostic reasoning based on measurable outcomes, such as blood pressure and blood counts. Theories also informed physician-speakers’ way of thinking of medications, differentiating drugs per category, such as curative and preventive drugs. Citizens did not address drugs as preventative, discussing drugs as mostly curative. Physician-speakers, however, also described limitations to biomedical knowledge. Research barriers limited the available evidence for elderly populations over 75 years of age, an area of knowledge that physician-speakers described as an "evidence-free zone." When discussing these limitations, physician-speakers described other types of knowledge as shaping their way of thinking, as illustrated in the sections below.

On the other hand, citizens expressed alternative views on what they understood as biomedical knowledge. Citizens described biomedical knowledge in terms of knowledge to make informed decisions, that is, to inform the rationale that defines the use and dosage of medications, especially for asymptomatic conditions. They expressed their knowledge of the causes, development and consequences of diseases as symptom-based knowledge, family history and healthy lifestyle behaviours, such as diets and physical activities. Most citizens expressed confusion about their own prescriptions due to the lack of symptoms, as this one citizen explained: “At 50, with menopause, I don’t know if it is related, bang, I have to take cholesterol medication, but I cried, I didn’t want medication, I was looking out for my health.” (Workshop 16)

They also discussed biomedical knowledge as evidence or content from individual studies they were knowledgeable about. However, they viewed scientific evidence also in terms of access and barriers to this knowledge and not only in terms of content. Citizens described interest in accessing the sources of scientific evidence presented during the workshop—e.g., the Compendium of Pharmaceuticals and Specialties and the Beers List—as well as how research is conducted and scientific evidence produced, as this member of the public probed: “Can a doctor prescribe a drug to a patient to collect data for a research project without telling the patient it’s for a project?" (Workshop 1)

#### Sociocultural beliefs

Participants shared several culturally and socially informed beliefs when discussing PIMs with the public during workshops. Participants voiced these beliefs as knowledge on medications, health, sickness, treatments, health professionals’ roles, and encounters in the clinical settings. Sociocultural beliefs were constructed as shared assumptions understood to be true without evidence and derived from localized direct and indirect experiences. Often, beliefs were expressed as anecdotal evidence, i.e., knowledge based on reports of individual cases rather than on systematic research or analysis. Physician-speakers’ anecdotal evidence derived from professional experiences in clinical settings, expert opinions that included medical expert consensus, consultation and clinical experience of other health professionals, and in a few instances from family and friends. Communication specialists-facilitators’ anecdotal evidence stemmed from their own experiences with the health care system and research, from their own professional experiences as scientific journalists and communicators, and the expert opinions of health professionals and scientific media. Citizens drew most of their anecdotal knowledge from health experiences with illnesses, medications and side effects, experiences accessing and navigating the health system and health care professionals, and experiences accessing and interacting with health care information, in some instances, also from personal, professional experiences as health care workers. Finally, citizens valued and often used other sources of knowledge beyond the health system, such as family, friends, traditional and online media. Physician-speakers and communication specialists-facilitators discussed a set of common beliefs shared among health providers and communication specialists-facilitators. They believed in scientific progress and its ability to improve health and health care naturally and organically over time. These participants also asserted beliefs in new clinical practices, such as shared decision-making (SDM) and deprescribing, as this one physician described: “In the past, doctors were more paternalistic and then (…) they are going to give more drugs. But now it’s much more (…) non-pharmacological. Then everything that is the Deprescribe movement, that is a national movement.” (Workshop 15)

However, they also indicated that these beliefs were not shared consistently among doctors. Similarly, physician-speakers and communication specialists-facilitators discussed beliefs as social and cultural trends shaping citizens’ expectations, knowledge, and behaviours.

Advertisement and food industries played an important role in shaping expectations among citizens, feeding off what physician-speakers and communication specialists-facilitators defined as a medicalized society, that is, a society organized around a common trust in medications’ beneficial effects. Physician-speakers discussed media’s influence in promoting misleading notions about medications, about specific types of health practices, such as diets, homeopathy, and consultation practices, e.g., turning to "Dr. Google" rather than to doctors to learn about their health status and treatment options. Whereas physician-speakers and communication specialists-facilitators focused on beliefs of scientific development and clinical practice innovation, citizens held differing beliefs. Citizens often voiced anti-medication views. Anti-medication beliefs often meant avoiding medications and preferring natural products or lifestyle behaviours. These beliefs also shaped citizens’ views and knowledge of health care professionals and the health care system. Some citizens believed doctors had all the answers to their questions and could fix all their health problems. Others believed physicians prescribed too many medications, or that physicians in academic clinical institutions were less qualified, or that pharmaceutical companies influenced physicians’ clinical and prescribing behaviours:

“I think that very often doctors are prescribing drugs [for] people 65 and older, but doctors are prescribing a lot and too much.” (Workshop 23)

#### Value-based knowledge

At the most abstract level, participants discussed ways of thinking based on values. Values are preferences, wishes, needs, positive and/or negative evaluations and judgments. Values represented what was important to individuals. All participants referred to both patients’ and physicians’ values.

Physician-speakers and communication specialists-facilitators explicitly and implicitly expressed that values influenced and shaped their thinking. They used value-based reasoning during the knowledge exchange sessions when discussing patients’ freedom of choice. Choice was framed as respecting patients’ autonomy, ensuring fair and transparent access to knowledge and fair decision-making in health care. Physician-speakers and communication specialists-facilitators discussed patients’ autonomy as respecting and empowering patients to gain a greater role and become active participants in health care, as this physician described: “It is always the patient who decides first.” (Workshop 21)

Concurrently, they framed patients’ freedom of choice as patients’ fair access to knowledge together with health care providers’ support, ensuring patients received clear and transparent knowledge for informed decision-making. Similarly, citizens shared the same understanding of freedom of choice as respecting patient autonomy, ensuring patient empowerment and partnership through education and fair participation in decision-making. Yet, citizens expressed these values by describing the lack of opportunities for patient empowerment and the lack of participation in health care decision-making. In other instances, some citizens expressed appreciation collaborating with physicians, and others stressed their active role in controlling their health, as this citizen emphasized:

“I think that we are the orchestra directors at the moment. Because in my case, that’s what I’m doing, I’m the one who goes from one to the other and asks to look at this or that thing.” (Workshop 7)

Citizens expressed the value of trustworthy sources of knowledge, both in and beyond the health system. Citizens discussed trustworthiness in terms of transparency and the legal and ethical conduct of health professionals. Some citizens voiced their trust and desire to trust their health professionals. Others expressed concerns regarding the integrity of health professionals’ recommendations, behaviours, roles. For example, citizens questioned pharmaceutical companies and physicians’ relationships and questioned pharmacists’ roles as both vendors and chemists interested in selling drugs rather than, or only, providing independent, trustworthy information. Citizens expressed worry and mistrust related to health care providers’ recommendations, as illustrated by this citizen: “At what moment, when a general practitioner gives us medication do we think whoa… because then I relied on my doctor and then… you know, I, I took some medication. Is there, uh, any place we can go to for information? I mean, how do I know that this, this, this doctor knows everything.” (Workshop 19b)

Citizens identified the need for transparent communication with their health providers as a condition for trust. Citizens valued clear instructions on the ways to navigate the health system–e.g., differences in accessing different clinical settings, such as family physicians versus hospitals, as well as clear and detailed explanations of their treatment alternatives:

“Speaking of options, I’ve never seen a doctor who said, uh, you have three options, and I’ll [give you the list here]. They say: Ok, you’re going to take this, you’re going to take this and you… I have… Do doctors now have to give us options?” (Workshop 19a)

Physician-speakers and communication specialists-facilitators also discussed trustworthiness as a value central to their way of thinking yet framing it in terms of credibility and accountability. First, they described scientific evidence as a source of accountability, often presented as opposed to online, non-health or non-research-related sources of knowledge. Second, they expressed these values as providing regular check-ups and medication audits as well as ensuring health providers’ and patients’ collaboration. Physician-speakers and communication specialist-facilitators also included values related to ethical principles inherent in clinical practice and professionalism. These participants described ethical principles of clinical decision-making, such as avoiding harms and delivering benefits, professional deontological code and medical training as essential forms of knowledge shaping their views and behaviours.

Credibility and accountability, together with medical education, law, professional and governmental authorities, such as the College of Physicians, were also discussed as established means to ensure protecting the public’s safety by regulating the practice of medicine through licensing policies, as this communication specialists-facilitator described: “You are dealing with a doctor, you are dealing with someone who is a member of a professional order. (…) we know all about the training he received, the courses, the uh… No, but all that when you deal with a naturopath, he has no professional order, and has no mandatory naturopathic training. You go there, uh, it’s a Russian roulette, eh? But no guarantee.” (Workshop 13)

Citizens shared physician-speakers and communication specialists-facilitators views on accountability as a relevant value and voicing the desire for care continuation and coordination, including audit and monitoring of their medication list and use of medications. Additionally, citizens described accountability also as defined roles and responsibilities. In the context of the conference topic—i.e., PIMs—this meant defining which physicians, specialists or family physicians, were responsible for prescribing and reviewing patients’ medications. Citizens discussed the government’s role and responsibilities, the market, pharmaceutical companies and insurance companies, addressing issues of accountability for selling over-the-counter drugs, ensuring that medications are regularly reviewed, and the government’s control over insurance companies.

#### Institutional knowledge

When discussing PIM, participants, especially citizens, expressed knowledge as understandings about the interplay of relationships among institutional actors and material resources embedded in practices, routines, and procedures. Participants spent time discussing institutional knowledge as the understanding of regulating and organizational frameworks that define the distribution, access, and use of health care resources. In particular, participants addressed knowledge as practical knowledge on accessing and navigating health care services. Citizens identified both barriers and facilitators in accessing and navigating the health care system. These participants described the shortage of family physicians and limited consultation time with providers as primary obstacles to care: “When you say: talk to your doctor. They don’t listen to us that much, and then they can’t wait for us to get out of the office. Uh, well, if he’s standing, he turns his back on us, and then we go, we’re going to have his back on us, no communication. I’m telling you, it’s a terrible thing to live through.” (Workshop 19a)

Yet, citizens also described being aware of alternative points of access to health care and knowledge, mostly indicating pharmacists as a common alternative to physicians: “But in fact, pharmacists are extremely open; they have, they are not in the rush of the appointment.” (Workshop 19a)

Citizens relied on family physicians and traditional sources of knowledge, including family, friends, and media, to learn to navigate the health system and access care. Physician-speakers and communication specialists-facilitators identified family physicians and pharmacists as primary sources of knowledge. They also discussed trustworthy sources of knowledge from traditional and online outlets, such as conferences, Doctissimo, Extenso, Passeport Santé, and tools, such as Carnet Santé Quebec and Choosing Wisely. However, citizens identified the lack of coordinated care and lack of knowledge and skills to navigate the health system as another barrier to health care and knowledge. They often described disagreement among different healthcare professionals as a cause of disorientation and uncertainty regarding which health recommendation to follow: “My question was how do you deal with a doctor, the one, the neurologist who wants to prescribe an Alzheimer’s drug, and the cardiologist who says: No, no, no, it makes tachycardia, he already has a low pulse.” (Workshop 7) Physician-speakers and communication specialists-facilitators discussed different health professionals’ roles and duties instead, describing the family doctor as the gatekeeper of care and the pharmacist as the medications’ expert. Concurrently, these participants indicated how health professionals collaborate and work in institutionalized frameworks aimed at coordinating teamwork: “Doctors these days, we work in FMGs, in family medicine groups, and when your doctor works there, sometimes you have access to a pharmacist (…) and geriatricians, who are specialists in geriatric problems. (…) Often your family doctor has access to many specialists, including a nurse including a pharmacist. Ask your doctor: do you have a pharmacist in the building? He might say no, he might say yes. Tell him you want to meet them. It’s not expensive for the doctor. The doctor writes a prescription and then says: perfect, follow up in the pharmacy.” (Workshop 18b)

When discussing the role of institutions, physician-speakers and communication specialists-facilitators talked about systemic barriers to care delivery. These participants viewed scarce resources and an overburdened health system limiting the availability of human healthcare resources, doctors’ time with patients and coverage of alternative medicine services. Citizens, on the other hand, did not consider systemic barriers. Instead, financial and medical insurance barriers related to health care and medication costs represented a matter of concern for them, as these matters affected their health and health care.

## Discussion

Our findings describe public libraries citizen workshops creating interactive dialogical spaces in which citizens, communication specialists, and health professionals draw upon different types of knowledge to understand and make sense of primary care research outcomes. We can make some parallel with functions of TV news such as reporting information to the public or “cultivating community values, beliefs, and norms” [55]. Although television may be widely used to communicate health information to the public, this medium does not enable direct interactions between the messengers and the recipients, which could limit the kinds of knowledge as well as the depth of the knowledge that the information seeker is looking for and what is communicated. When discussing primary care research outcomes on PIMs, the workshop participants considered both evidence-based and non-evidence-based forms of knowledge. Health professionals and communication specialists tended to prioritize biomedical, evidence-based forms of knowledge while acknowledging its limitations and valuing sociocultural beliefs, values, and institutional forms of knowledge. Similarly, citizens valued biomedical knowledge, yet they understood knowledge as contextual, non-reductionist, and connected to social and material issues of access to health knowledge and care. Citizens seemed to prefer knowledge that was accessible, trustworthy, and left room for action and ownership over their understandings. This aligns with previous research describing the value of providing research outcomes to the targeted user group that are action-oriented [56]. For example, citizens viewed biomedical scientific knowledge not only in terms of content but also in terms of accessing and using such knowledge.

As others have noted, these findings suggest that access to primary care outcomes is necessary and needed, but also that making such information meaningful to citizens by identifying what type of knowledge informs a meaningful interpretation [57]. As previous studies have described [58,59], our findings tell us that different knowledge users are likely to understand and make sense of primary care research outcomes through the lenses of diverse scientific, normative, material, and institutional knowledge systems.

Similar to our findings, but in the context of public health program planning and knowledge exchange, authors of another study found that public health workers used explicit, evidence-based and tacit forms of knowledge, drawn from experience, context-specific and subconsciously understood knowledge to guide program planning [58,59]. Our study contributes to KT’s field by showing that a broad spectrum of knowledge systems characterizes knowledge exchange interventions targeting citizens. Our work adds to the increasing acknowledgment that mobilizing evidence into practice is a "complex process that involves different disciplinary approaches, beliefs, values and worldviews" [58].

Our study’s findings indicate that the scope of knowledge dissemination and exchange activities with citizens in public settings should include other types of knowledge along with formal research outcomes. Finally, this study highlights how public libraries enable to create interactive dialogical spaces in which citizens can learn and share knowledge in a meaningful way. However, while public libraries usually offer in-person services, workshops and events, and the citizen workshops have followed this traditional practice, this may not always be possible. For example, recently, the world has been hit by an unprecedented pandemic, i.e., COVID-19, changing how individuals meet and gather in public spaces. For the time being, COVID-19 related physical distancing measures suggest that public libraries are not ideal places for in-person interventions, thus, limiting in-person citizen workshops. While in-person workshops are valuable for the interaction among participants, ensuring equal access and interaction in these dialogues on online programs can be a challenge. Many may not have the means and skills to connect and actively participate in the dialogues via the internet and computer devices. However, considering the current pandemic and physical-distancing measures, as well as limited access to in-person workshops for elderly who have limited mobility and their caregivers, future research should investigate online or hybrid online/in-person knowledge exchange interventions. For example, investigating online learning platforms that allow active interaction among participants and examine other benefits of virtual interactive knowledge exchange programs, such as greater reach and participation. Perhaps, still using public libraries. In Quebec, for example, public libraries demonstrated to play an essential role in their communities even during the Covid-19 pandemic [60]. Through the trust public libraries generate among the public, they can offer in-person and online programs to disseminate and exchange research knowledge to the public.

This study also has some limitations. First, this study was primarily limited by its design. As a secondary analysis, we were restricted in the content and focus of the existing recorded data. However, we tried to increase the study’s trustworthiness by regularly peer debriefing with the parent study research team. The parent study research team, put together using the IKT framework, was comprised of various stakeholders–i.e., patient partners, librarians, physicians and researchers. These team members were very familiar with the citizen workshops, the collected data and provided valuable insights to contextualize the data during the analysis. We do acknowledge that the parent study adopted a convenience sampling strategy to recruit citizens and ensure a wide variety of participants and perspectives. However, the way the intervention was advertised, scheduled and located—i.e., public libraries—resulted in a skewed sample comprised of older, mostly well-educated women. There is likely of self-selection in that participants had an interest in learning about the topic, and the quantitative findings from the parent study suggest that there was a notable knowledge gain from participants [25]. Participants were indeed, to some extent, highly opinionated and self-aware of their health and health care, directing the discussions often on matters of chronic condition management and elderly care. We believe that a reason for high participation of elderly individuals might have been because of the topic, i.e., PIMs among the elderly, and time of the day in which the workshops took place, i.e., during regular working hours. We also believe that the advertisement channels might have contributed to citizens’ self-selection, which targets a more mature and highly educated audience. However, the audience’s demographic characteristics are consistent with previous research showing that citizens from lower socioeconomic strata and lower literacy tend to seek and use less health information than individuals with a higher socioeconomic status and education. [61–64]; Reimer-Kirkham & Jule, 2015) This sample also agrees with previous studies examining gender differences in information-seeking behaviours, indicating that women generally seek knowledge more than men [65,66]. Further dissemination and exchange interventions with the public should pay careful attention to target male and low socioeconomic status citizens to ensure that primary research outcomes reach and benefit all segments of the public. Second, data saturation was discussed during the analysis. However even through a rich descriptive database was available and we deemed the data rich and thick enough to establish saturation, our analysis and conceptual development was restricted in content and focus by the existing recorded data. The workshops were all on one specific topic, i.e., PIM, which could question the generalizability of our results to other health topics. Despite that some comments or quotes from participants were specific to the topic, we identified kinds of knowledge (biomedical, sociocultural beliefs, value-based reasoning, and institutional knowledge) that could apply to other general health topics and that are consistent with those of other studies [56–59]. The topic of PIM is not central to our study as we aimed to examine the knowledge transfer process in a neutral environment (a public library). Hence, the topic was selected based on the consideration of being of interest to as many people as possible. This larger study was also implemented after a successful pilot [24] which reported positive results in terms of knowledge gained by participants.

Finally, this study includes secondary post hoc analysis based on transcripts derived from the parent study. It does not include other empirical data collection and analytical techniques, such as individual interviews or document analysis, which can contribute to a richer understanding of the meaning-making process developed during the workshops’ discussion sessions. This study will then be most helpful in identifying the essential dimensions of knowledge in KT processes among physician-speakers, communication specialists, and citizens crucial for future investigation and implementation. Future research should consider examining individual participants’ perspectives and experiences of participating in a knowledge exchange activity in public settings. This study then can offer essential insights to build a new theory in the field of knowledge transfer practices that target the public.

This study has also strengths. Because knowledge exchange activities with the public are still new and mostly unexplored, the expected findings will contribute to knowledge and practice in different ways. First, secondary post hoc analysis offers two important advantages. It allows to ask new questions that were not of interest to the original investigators [67]. Second, we considered the data generated in the parent study as an opportunity to have access to data that would otherwise be difficult to collect. The parent study intervention is one of the first and only studies to design and implement a large-scale KT intervention with citizens in public settings. Most literature on knowledge transfer generally focuses on four groups of users, i.e., researchers, health care providers, decision-makers, and patients in the clinical setting. Little to no knowledge has been researched or discussed on knowledge dissemination and exchange practices with the public in public settings. As such, this study offers an essential contribution in examining the shift from siloed knowledge transfer research and practices to large-scale audiences benefiting a greater number of individuals.

Finally, this work also contributes to the larger KT picture and its practice within public settings with citizens by offering insights into the multifaceted nature of knowledge. This study’s findings bring to the attention the value-based, socio-culturally determined beliefs and institutional knowledge systems that—together with scientific evidence—construct knowledge that can inform dissemination and knowledge exchange interventions with the public. As these knowledge systems appear to play a relevant role in making sense and implementing evidence-based knowledge for practice and policy [58], identifying and describing them will help build a shared understanding among different stakeholders about the types of knowledge used and valued future dissemination and knowledge exchange interventions.

## Conclusions

Research evidence does not speak for itself. Researchers need to actively mobilize such evidence to ensure it benefits its users, i.e., citizens [2,68]. One way to do so is through citizen workshops in public settings. These workshops can be understood as a suitable space for connecting researchers, health professionals, and members of the public, opening interactive dialogical spaces that can help bridge research with its users and benefit citizens at large. We hope that this project will lay the groundwork to develop dissemination and knowledge exchange strategies that will facilitate the dissemination of research results to the public through innovative and promising communication channels beyond the clinical consultation setting.

## Abbreviations

FMG: Family Medicine Group
IKT: Integrated knowledge translation
KT: Knowledge transfer
PIMs: Potentially inappropriate medications
SDM: Shared decision-making
